# Sex Differences in the Effects of Cognitive Reappraisal Training on Conditioned Fear Responses

**DOI:** 10.3390/ijerph192315837

**Published:** 2022-11-28

**Authors:** Suqun Liao, Wen Xiao, Yancai Wang

**Affiliations:** Teacher Education School, Shaoguan University, Shaoguan 512005, China

**Keywords:** cognitive reappraisal, conditioned fear, acquisition, extinction, sex difference

## Abstract

Sex differences in emotion regulation strategies may impact sex differences in affective disorders. Using cognitive reappraisal strategy in the discriminative task of conditioned fear was studied to understand how sex differences in emotion regulation impact on conditioned fear in men and women. College students with low cognitive reappraisal scores completed the task of conditioned fear during two days: acquisition and extinction at the first day, and re-extinction at the second day. The reappraisal training was carried out before conditioned fear task. The self-reported fear rating of the conditioned stimulus (CS) and US-expectancy in the conditioned fear (unconditioned stimulus, US) were analyzed. Results showed all subjects acquired conditional fear and successfully distinguished CS+ from CS−. Cognitive reappraisal significantly reduces the fear rating and improves the extinction of US-expectancy in both sexes, but the fear rating in female reappraisal group decreases more slowly than that in male reappraisal group, as well as the extinction of US-expectancy in woman requiring a longer time and more trials of extinction than that in men. For individuals with low cognitive reappraisal scores, cognitive reappraisal promotes the extinction of conditioned fear in both males and females. Because of the original gender difference of conditioned fear extinction and emotion regulation, the effect of cognitive reappraisal on conditioned fear is complex, which shows differently in influence speed and practice effect.

## 1. Introduction

In terms of emotional regulation, women are generally more prone to anxiety and depression than men [1,2]. Previous works have indicated that women are more likely to experience anxiety and depression symptoms due to stronger emotional responses to negative stimuli and inappropriate emotional regulation modes [3,4]. However, in-depth research on affective disorders has shown that improper emotion regulation redoes not have a substantial impact on various mental diseases [5,6]. Therefore, sex differences in affective disorders, especially emotion regulation strategies, should be investigated [6,7,8,9].

Cognitive reappraisal is an important emotion regulation strategy that involves reinterpreting negative emotional stimuli according to different scenarios and perspectives to change one’s mood [10,11,12,13,14]. Previous studies have shown that cognitive reappraisal is effective for addressing adverse events, sustainably regulating negative emotions, and reducing anxiety and depressive symptoms. Previous meta-analyses have shown that cognitive reappraisal changes connections between stimuli and emotions. After cognitive reappraisal, self-reported negative emotions decreased, while positive emotions increased [15], with heart rate and skin potential levels effectively reduced [16]. A study on adolescent students by Duarte et al. showed that cognitive reappraisal reduced anxiety and depressive symptoms in both sexes [17]. The above studies suggest that cognitive reappraisal is an effective positive emotion regulation strategy for both sexes and different age groups. However, sex differences in cognitive reappraisal and the exact relationship between cognitive reappraisal emotional regulation strategies and sex differences in affective disorders, such as depression and anxiety, remain unclear.

The conditioned fear process, which is based on Pavlov’s conditioned reflex theory, includes the acquisition, storage, retrieval and extinction of fear memory, and this process is often used to explain the pathological development of anxiety disorders, phobias, PTSD, and other affective disorders. Moreover, conditioned fear responses have been used to predict susceptibility to pathological anxiety and fear. Compared with healthy individuals, patients with anxiety disorders exhibited stronger fear responses more quickly, as reflected by stronger skin conductance responses and higher fear scores during the acquisition and extinction of conditioned fear, thus showing delayed fear extinction or extinction disorders.

Few studies have investigated sex differences in conditioned fear memory in healthy populations. If emotion regulation strategies are ignored, conditioned fear tasks may also indicate certain sex differences; however, the results vary. Fredrikson et al. implemented a discriminative conditioned fear paradigm with dangerous animals as the conditioned stimulus (CS) and electrical stimulation as the unconditioned stimulus (US) and found no significant differences in the acquisition and extinction of conditioned fear between the two sexes [18]. Zeng Qing and Zheng Xifu compared sex differences in the acquisition of fear, disgust, and neutral emotions, and found no differences between the two sexes. Sun Nan and Zheng Xifu used a conditioned fear paradigm [19] to study the time course of event-related potential changes in gender-related learning, believing that it was inappropriate to draw the simple conclusion that men acquire fear memories more easily or that women have more difficulty addressing fear because neural activity differences may occur in various time courses [20].

Previous studies have suggested that fear memory is influenced by physiological and biological structure functions, social culture, and individual cognitive and emotion regulation; however, few studies have reported whether individual emotion regulation strategies affect fear memory acquisition and extinction more than other factors. Blechert et al. presented subjects with a brief 5 min cognitive reappraisal strategy before conducting experiments. In a discriminative conditioned fear task, three CSs were designed, including two CS+ and one CS−. The subjects were instructed to selectively reassess only one CS− during the acquisition and extinction stages of the discriminative conditioned fear task. The results showed that the reappraisal CS− of female subjects extinguished faster than the non-reappraisal CS−, implying that women may follow cognitive reappraisal instructions better than men [21]. However, this study had several limitations. For example, in the acquisition stage, the number of male subjects was less than half of the number of female subjects. Therefore, how sex differences impact the use of cognitive reappraisal as an emotional strategy against conditioned fear merits further study. Short-term reappraisal guidance has a limited duration [21]. Although longer reappraisal training can effectively promote the extinction and return of conditioned fear [22,23,24], it remains unclear whether there are any differences between short-term and long-term training.

This study combined positive emotion regulation strategies with conditioned fear paradigm in individuals with low cognitive reappraisal scores, to reveal sex differences in the effects of cognitive reappraisal training on conditioned fear response. This was helpful to better understand the physiological and psychological mechanisms [25] underlying sex differences in susceptibility to mental disorders, and to provide references for improving and developing psychotherapy methods for gender-specific clinical anxiety disorders, phobias, and other disorders.

## 2. Materials and Methods

### 2.1. Participants

In this study, the G* power 3.1 software [26] was used to calculate the sample numbers. The effect size was set as 0.25 and α setting was 0.05. The calculation results show that 76 subjects are required for the study in order to reach the statistical test force of 0.95. 119 subjects (58 males and 61 females) were actually recruited aged between 18 and 24 years. The subjects were all right-handed, had no history of physical or mental disease, were not color blind or color weak, had normal or corrected-to-normal vision, and had no fear of the color red, blood, or nausea. The menstrual cycles of the included females ranged from 21–35 days, and the subjects were not in their menstrual period during the experiments. The recruited subjects voluntarily participated in the experiment, and a reward was provided after the experiment. Informed consent was signed prior to the start of the experiment.

The emotion regulation strategies of college students were measured with the GROSS self-rating questionnaire on Emotional Regulation Styles including 6 cognitive reassessment categories, which total score was 42 points. The lower the score, the less likely they are good at using cognitive reappraisal for emotional regulation. Students with a total score of less than 24 are considered as individuals with low cognitive reappraisal. 752 college students were investigated and 169 students with low cognitive reappraisal scores were selected, in which 131 students volunteered to participate in the experiment. The participants were randomly divided into experimental group and control group according to gender. If the following conditions occurred during or after the experiment, the participants’ data would be deleted: (1) affected by the environment during the experimental process; and (2) within 5–7 days after the end of the experiment, the female subjects reported that they experienced emotional symptoms such as anxiety, irritability or other physical discomfort 5–7 days before the menstrual period. The final data was from 119 participants, of whom 61 people were in the reassessment group (30 males and 31 females) and 58 people were in the control group (28 males and 30 females).

### 2.2. Experimental Apparatus and Materials

#### 2.2.1. Stimuli

The experimental device was a Lenovo notebook with a 13-inch display, a resolution of 1024 × 768 and a refresh rate of 85 Hz. The experimental presentation was compiled by E-Prime 2.0. The CS and US used in the discriminative conditioned fear task were both images from the International Emotional Picture Library. The CS was two images of tomatoes numbered 7285 and 7351 in the library, and the US was a traumatic image, numbered 3068, 3010, 3005.1, 3000, 3071, 3080, 3102, or 3150 in the library, with a valence of 1.74 ± 0.23 and an arousal of 6.89 ± 0.23.

#### 2.2.2. US-Expectancy Measure

Following CS presentation, participants rated their expectation of the US. The question “Is there a negative picture?” was presented using a 10-point scale, from 0 (certainly no negative picture) to 9 (certainly a negative picture). Participants rated their expectancy of a negative affective sound by pressing the corresponding number key.

#### 2.2.3. CS Fear Ratings

All participants were required to rate the valance of the CS+ after acquisition, revaluation, and extinction on a 9-point scale from 1 (no pleasure) to 9 (very much pleasure). This scale is designed to assess the degree of fear participants elicited by CS+.

### 2.3. Positive Cognitive Reappraisal Training

The reappraisal training in this study was based on the reappraisal training method proposed by Shurick et al. [22]. One day before the experiment, the subjects were trained on the positive cognitive reappraisal. The reappraisal training consisted of 4 steps. (1) First, subjects were trained to understand that different ideas about a thing lead to different emotions. For example, about a picture of “a person is lying on a hospital bed”, thoughts of “he is filming” would not lead to a negative emotion, while thoughts of “This is my close relative, he is seriously ill, and the disease has tortured him to death” might lead very much to sadness. (2) Through group discussions and sharing, subjects strengthened their beliefs that different thoughts about the same image or event can produce positive emotions and investigated methods that cause such beliefs. Each group included 6–10 people, with a group leader. Each group was provided with negative images for group discussion, and each group member shared their most effective method for reducing negative emotions and improving positive emotions for recognition. (3) During the test step, the group leader presented some pictures and asked the subjects to positively understand, one by one, which made sure each subject learned to reduce negative emotion by positive cognition of negative pictures. (4) Consolidate. The subjects were asked to reappraise the events, people, scenes, etc. that they encountered in their daily lives before the end of the formal experiment. Moreover, before the formal experiment, the subjects would be checked in their reappraisal; those who failed the test, would not be allowed to participate in the experiment.

### 2.4. Experimental Design and Procedure

The conditioned fear task had two stages: acquisition and extinction, which adopted the discriminative conditioned fear experimental paradigm, including two CSs, namely, CS+ and CS−. The CS were presented in randomized order, with the restriction that no more than two subsequent trials could be undertaken.

The experimental procedure adopted a block design with three stages: habituation, acquisition, and extinction [27]. The CSs appeared sequentially on a screen with a presentation time of 8000 ms, and the US or white screen appeared with a presentation time of 6000 ms and an intertrial interval (ITI) of 16~20 s.

At the habituation stage, CS+ and CS− were randomly presented in 3 trials. At the end of each trial, the subjects were asked to give the US-expectancy on a 9-point rating scale that ranged from 1 to 9. Before, and at the end of the phase, the subjects were asked to orally report the fear valence of CS with a number that ranged from 0 (no fear) to 100 (a lot of fear).

In the acquisition stage, CS+ and CS− each appeared in 8 trials with CS offset always immediately followed by the US (reinforcement rate 100%). The forecast of US expected value and the verbal rating of CS were identical to the task at the end of habituation.

The extinction stage included two parts: 5 min after the end of the acquisition stage (extinction) and 24 h later after the first extinction (re-extinction). The two extinctions were exactly the same with CS+ and CS− each appearing in 12 trials. The forecast of US expected value and the verbal rating of CS were identical to the task at habituation stage.

### 2.5. Data Analysis

All analyses were performed with SPSS 24.0 (Chicago, IL, USA). CS+ fear rating and US-expectancy were analyzed using a mixed analysis of variance (ANOVA) for repeated measures with the groups and the gender as the between-subjects factor, and trial (i.e., stimulus presentation) as within-subjects factors. The US-expectancy were repetitive ANOVA in extinction stage between reappraisal group and control group with the sex as the between-subjects factor, and stimulus (CS− vs. CS+) and trial (i.e., stimulus presentation) as within-subjects factors. Significance level was set at *p* < 0.05.

## 3. Results

### 3.1. Fear Rating

A repeated measures ANOVA indicated the mean CS+ fear rating significantly increased from post-acquisition to post-extinction between the two groups showed a significant main effect of group (F(1,118) = 11.31, *p* < 0.01, *η*^2^*_p_* = 0.121) and a significant interaction effect between group and gender (F(1,118) = 22.32, *p* < 0.001, *η*^2^*_p_* = 0.242). Simple effect analysis showed that the fear scores of CS+ in the reappraisal group were lower after acquisition (M control = 81.82 ± 21.43; M reappraisal group = 71.51 ± 21.32; T (116) = 10.32, *p* < 0.01), extinction (M control = 52.17 ± 14.76; M reappraisal group = 46.94 ± 12.67; T(116) = 3.32, *p* = 0.075), and re-extinction (M control = 32.18 ± 9.92; M reappraisal group = 24.14 ± 9.98; T(116) = 6.33, *p* < 0.05). 

**Figure 1:**
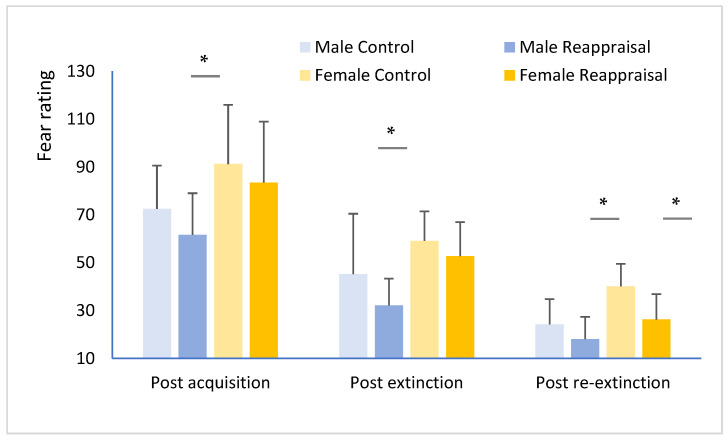
CS+ fear ratings. Post acquisition, post extinction, and post re-extinction, for the reappraisal and control groups * *p* < 0.05.

An independent samples t-test for CS+ was conducted to assess differences between the male and female. The CS+ fear scores of men’s fear scores were lower than those of women, which showed differences changes at different task stages after reappraisal training (Figure 1). The CS+ fear scores were greatly lower after acquisition (M male control = 72.43 ± 18.13, M male reappraisal = 61.57 ± 17.42, T(56) = 8.33, *p* = 0.012) and extinction (M male control = 45.21 ± 28.23, M male reappraisal = 32.15 ± 11.20, T(56) = 8.56, *p* = 0.011)) in men reappraisal, while which was significantly lower only after the re-extinction stage in female (M female control = 40.11 ± 9.35, M female reappraisal = 26.25 ± 10.05, T(59) = 8.21, *p* = 0.015). The above results showed that the conditioned fear rating was slowly decreased in woman experiencing reappraisal training from extinction to the end of re-extinction stage, which differs from the conditioned fear rating quickly decreasing in men after acquisition.

### 3.2. US Expectancy

#### 3.2.1. Acquisition

**Figure 2:**
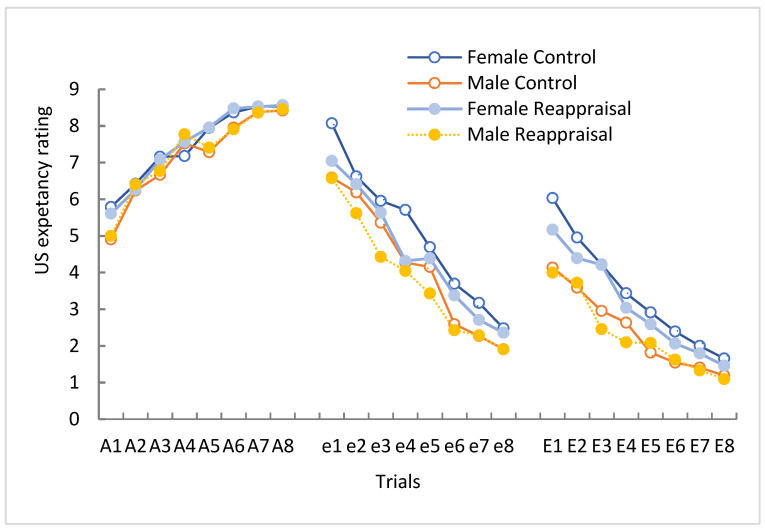
Mean expectancy rating of CS+. The US-expectancy of the CS+ during acquisition (A1–A8), extinction (e1–e8) and re-extinction (E1–E8) for the devaluation and control groups.

A trial* CS type*group repeated measures ANOVA of US expectancy ratings revealed no differences between control and reappraisal group during acquisition stage (F(1,1) = 1.06, *p* > 0.05, *η*^2^*_p_* = 0.0223). The main effects of CS types was significant difference (F(1,1) = 2367.847, *p* = 0.00, *η*^2^*_p_* = 0.889), as well as significant main effects of trials (F(1,7) = 51.65, *p* = 0.00, *η*^2^*_p_* = 0.432). There was a significant interaction between CS type and trial (F(1,1) = 26.72, *p* < 0.001, *η*^2^*_p_* = 0.325) characterized by a higher expectancy rating for the CS+ than for the CS− trials (Figure 2). The data indicated that participants learned to expect the US on CS+ trials and not to expect the US on CS− trails during acquisition, and that all participants acquired conditioned fear and distinguished CS+ from CS− successfully. 

#### 3.2.2. Extinction

The US-expectancy rating decreased faster in the reappraisal group (Figure 2). Repeated measures ANOVA of CS+ US-expectancy rating during the extinction stage showed significant main effect of groups (F(1,1) = 4.14, *p* = 0.041, *η*^2^*_p_* = 0.091) and of gender (F(1,1) = 34.076, *p* = 0.000, *η*^2^*_p_* = 0.421), as well as significant interaction main effects of group * gender (F(1,118) = 3.873, *p* = 0.046, *η*^2^*_p_* = 0.082). Respective univariate ANOVA of CS+ US-expectancy rating of trials (e1–e8) in men and women revealed gender differences in the effect of cognitive reappraisal on CS+ extinction. The group main effect between reappraisal group and control group was significant in women (F (1,490) = 3.967, *p* = 0.0450, *η*^2^*_p_* = 0.084), but there was no significant difference in men (F(1,526) = 2.597, *p* = 0.067, *η*^2^*_p_* = 0.022). The above results showed that reappraisal had greatly decreased the CS+ US-expectancy rating of female than that of male.

A trial *gender *group repeated measures ANOVA of CS+ US-expectancy ratings showed significant gender main effect (F(1,1) = 46.97, *p* = 0.000, *η*^2^*_p_* = 0.476) and marginal significant group main effect in group main effect (F(1,1) = 3.867, *p* = 0.050, *η*^2^*_p_* = 0.088) during re-extinction stage. Respective univariate ANOVA of CS+ US-expectancy rating of different trials (E1–E8) in men and women showed significant difference between control group and reappraisal group just in female (F female (1,502) = 4.022, *p* = 0.042, *η*^2^*_p_* = 0.112; F male (1,502) = 0.034, *p* = 0.95, *η*^2^*_p_* = 0.002). So, compared with women, the CS+ US-expectancy ratings in men was degraded greatly than that in women, regardless of whether they had experienced reappraisal training or not.

**Figure 3:**
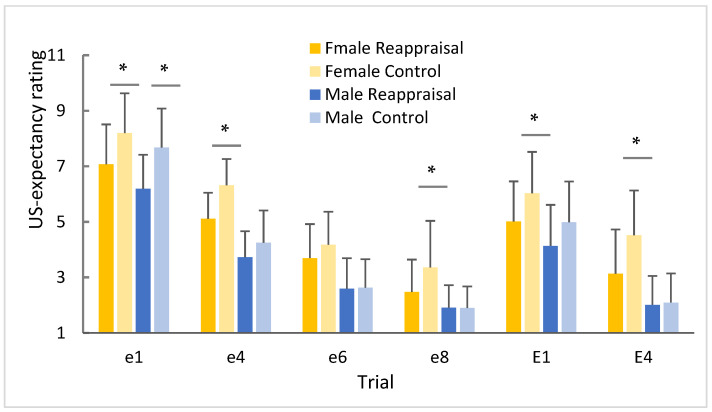
Changes of CS+ US-expectancy rating during different extinction trials. Note: e indicates the first extinction, and E indicates the second extinction after 24 h * *p* < 0.05.

Although US-expectancy showed no significant difference between the sexes after two extinctions, there were some obviously different changes during the extinction process (Figure 3). Compared with the control group, the CS+ US-expectancy of women in the reappraisal group decreased obviously and gradually in the first half of the extinction (T 1st trial (39) = 2.238, *p* = 0.032; T 4st trial (39) = 2.607, *p* = 0.013; T 8st trial (39) = 2.132, *p* = 0.043), while the CS+ US-expectancy in male reappraisal group were significant lower in first 4 trials (T 1st trial (39) = 2.689, *p* = 0.025; T 4st trial (39) = 2.08, *p* = 0.054).

During the re-extinction stage, the male reappraisal group was significantly lower than the control group only in the first trial, while the female was significantly lower in the first four trials. The above results suggested that, for women, cognitive reappraisal improved the extinction of conditioned fear and might need more practice.

The above results show that for individuals with low cognitive reappraisal scores, cognitive reappraisal promotes the extinction of conditioned fear in both males and females; however, compared with men, cognitive reappraisal improved the extinction of conditioned fear and might need more practice in females.

## 4. Discussion

In the present study, reappraisal training reduced the fear rating in individuals with low cognitive reappraisal scores and significantly improved the extinction of conditioned fear [19,22,28]. Regardless of the cognitive reappraisal, compared with women, the extinction of conditioned fear was faster in man [29]. Cognitive reappraisal seems to have a more obvious effect on the extinction of conditioned fear in women, but this process requires more extinction practice.

### 4.1. Cognitive Reappraisal Significantly Reduces the Negative Valence of Acquired Fear in Females

During the acquisition stage, all subjects showed significantly higher fear scores, indicating that reappraisal training did not inhibit the acquisition of conditioned fear in individuals with low cognitive reappraisal scores. However, regardless of sex, individuals who participated in cognitive reappraisal training showed significantly lower acquired CS fear scores than individuals in the control group. Moreover, the subjects had significantly lower CS fear scores in the extinction and re-extinction stages after 24 h, indicating that cognitive reappraisal is an emotion regulation strategy that may help individuals gain long-term emotion regulation effects.

Cognitive reappraisal reduces the negative emotional valence of conditioned fear, according to previous findings in behavioral and brain imaging studies. In a conditioned fear study, Hermannn et al. found that in aversive social scenarios, individuals with high cognitive reappraisal ability reported lower fear scores, decreased activation of the anterior cingulate and dorsomedial prefrontal cortex, and enhanced ventromedial cortical activation during the extinction stage [23]. According to the mental model of reappraisal, the reappraisal of aversive stimuli inhibited activity in the amygdala and insular lobe, which alters perceptual representations of aversive stimuli by changing the emotional meaning of the stimulus signals [25]. During conditional assessment of the CS, reappraisal updated the US mental representation by adjusting an individual’s perceptual representation of the aversive US [24,25]. According to the overall and reference hypotheses of CS valence formation, during conditional assessments, individuals encode CS and US simultaneously, and the reappraisal-corrected US mental representation reduces negative valence values, with the reduced negative valence transferred to the CS [27].

The scores of conditioned fear in men were significantly lower, which might be due to the sex difference in sensitivity to stimulus materials [30]. Fear rating was much lower in the females who experienced reappraisal training, which indicates reappraisal training more significantly affected negative emotional valence in woman.

### 4.2. Cognitive Reappraisal Rapidly Improves the Extinction of Conditioned Fear in Men

Cognitive reappraisal training enhanced conditioned fear extinction effects in individuals with low cognitive reappraisal scores. The dual neural pathway theory suggests that two kinds of memories are formed during the acquisition and extinction of conditioned fear: a fear memory of CS−US and an extinction memory of CS−no US. Cognitive reappraisal helps in the reconstruction of US representations. In the extinction stage, although the CS is not presented, CS may activate US mental representations and lead to the construction of new US representations, thus reinforcing the non-fear valence of the US [31].

The results in this study show the extinction of US-expectancy in reappraisal group males need the least number of trials, which suggest that reappraisal affected men more directly and quickly. The results are similar to those of related studies.

It is believed that men and women adopt different emotion regulation strategies in various situations. Under acute stress, men showed better cognitive reappraisal effects than women [32]. The physiological mechanisms may involve different hormone secretion processes and brain activity patterns in the two sexes. The glucocorticoid-driven mechanism suggests that acute stress induces cortisol secretion in men, thus enhancing the cognitive participation process. Stress-induced cortisol secretion has not been observed in women in the luteal phase or women taking contraceptives. Glucocorticoids are believed to have anxiolytic effects [33]. Glucocorticoids rapidly inhibited the response of the amygdala to negative emotional stimuli and that the abrupt withdrawal of glucocorticoids triggered selective attention to danger signals [32].

### 4.3. Cognitive Reappraisal Improving the Extinction of Conditioned Fear in Woman Needs More Practice

The complete extinction of US-expectancy of women in the reappraisal group needs more trials, both at extinction stage and re-extinction stage, which indicates that cognitive reappraisal improving the extinction in women with low cognitive reappraisal scores shows delay and depends on more practice trials. In men, the amygdala activity related to emotional response is decreasing [34], while activity in prefrontal activity related to cognitive reappraisal is less increased, which is thought to reflect the effort and frequency of conscious control for a given activity [33]. Thus, men incur less cognitive costs in cognitive reappraisal, possibly due to the initiation of automatic emotion regulation processes, which leads to their brains responding more quickly to cognitive reappraisal instructions [35].

Excessive stress immediately inhibits processes in the prefrontal control and impairs cognitive regulation of fear [32]. Meanwhile, stress promotes cognitive reappraisal processes in men to compensate for the inhibition of prefrontal activity and executive functions, thus effectively reducing fear responses [35]. Women have weaker cortisol responses to stress than men, possibly due to a complex interplay between glucocorticoids and glucocorticoid-dependent activity deficiencies in the prefrontal and marginal regions in women [32]. However, presenting the trials without US repeatedly during the extinction stage strengthen the work memory of CS-no US extinction memory obtaining long-term extinction.

Although this study revealed the sex differences in the effects of cognitive reappraisal training on conditioned fear responses, to some extent, it cannot be determined whether cognitive reappraisal is more effective at extinguishing negative emotions in men or women because great differences in cognition, emotion, and behavior existing in the two sexes [1,36]. Emotion regulating by cognitive reappraisal is a complex and interactive process of biological factors, psychological factors, and environmental factors [37,38,39,40].

This result is helpful to understand the pathological mechanism of gender emotional disorder and give some reference for improving the treatment of male and female patients with emotional disorders in clinical settings [41]. For example, careful selection of materials in exposure therapy may be needed because of women’s emotional susceptibility, and cognitive therapy for women patients may require longer clinical treatment time for their additional trials to complete extinction. However, due to the limitations of research objects and methods, it is necessary to use various technologies to reveal gender differences in emotional cognitive regulation in different groups.

## 5. Conclusions

Cognitive reappraisal cannot block the acquisition of conditioned fear regardless of being male or female, in individual low reappraisal scores. Cognitive reappraisal significantly reduces the fear rating and improves the extinction of US-expectancy in the two sexes, but the fear rating in the female reappraisal group decreases more slowly than that in the male reappraisal group; additionally, the extinction of US-expectancy in women requires a longer time, and more trials, compared to that of extinction in men.

## Data Availability

The datasets generated for this study are available on request to the corresponding author.
